# Propofol exerts anti-hepatocellular carcinoma by microvesicle-mediated transfer of miR-142-3p from macrophage to cancer cells

**DOI:** 10.1186/s12967-014-0279-x

**Published:** 2014-10-09

**Authors:** Jian Zhang, Wei-feng Shan, Te-te Jin, Guo-qing Wu, Xiao-xing Xiong, Hai-yan Jin, Sheng-mei Zhu

**Affiliations:** Department of Anesthesiology, The First Affiliated Hospital, College of Medicine, Zhejiang University, 79 Qingchun Road, Hangzhou, 310003 People’s Republic of China; Department of Oncology, Zhejiang Provincial People’s Hospital, Hangzhou, China; Department of Anesthesiology, The Children’s Hospital, College of Medicine, Zhejiang University, Hangzhou, 310003 China

**Keywords:** Propofol, Hepatocellular carcinoma, Microvesicles, RAC1, miR-142-3p

## Abstract

**Objective:**

We previously confirmed that propofol directly inhibited the viability, proliferation, and invasiveness of hepatocellular carcinoma cells *in vitro*. In this study, we investigated the mechanism underlying the anti-HCC effects of propofol.

**Methods:**

In vivo antitumor activity was investigated in tumor-bearing mice following an intraperitoneal injection of propofol, with or without clodrolip. The co-culture system was used to verify that miR-142-3p was transported from macrophages to HCC cells. A miR-142-3p inhibitor was used to down-regulate the expression of miR-142-3p.

**Results:**

Propofol drastically inhibited tumor growth in tomor-bearing mice through macrophage activation, and stimulated tumor-associated macrophages (TAMs) to secrete microvesicles (MVs), which delivered miR-142-3p to HCC cells, resulting in the inhibition of HCC cell invasion. In addition, MVs collected from the plasma of the tumor-bearing mice injected with propofol suppressed tumor growth. More importantly, down-regulation of the expression miR-142-3p reversed the effect of propofol on HCC cell migration.

**Conclusions:**

This study reveals a novel role for propofol in the inhibition of HCC through MV-mediated transfer of miR-142-3p from macrophages to cancer cells *in vivo*.

## Introduction

Hepatocellular carcinoma (HCC), a primary malignant tumor, has a poor prognosis and is the third cause of tumor-related deaths in China [1,2]. Although multiple treatment strategies, including surgical resection, liver transplantation, systemic or infusional chemotherapy, and targeted immunotherapy, have been applied, the overall 5-year survival rate for patients with HCC is still low [3,4]. Due to malignant proliferation and metastasis, there is no treatment to improve the survival of patients with advanced HCC [5,6]. Therefore, it is urgent to establish novel anticancer reagents to prevent the invasiveness of HCC.

Propofol (2, 6-diisopropylphenol), an intravenous anesthetic, also has antitumor effects [7,8]. Clinically relevant concentrations of propofol can induce apoptosis and inhibit the invasion of human cancer cells [9]. For example, propofol treatment resulted in the apoptosis of human promyelocytic leukemia cells [10]. It also significantly decreased the invasive activity of human colon carcinoma cells [11]. In animal studies, propofol appeared to exert antitumor activity by modulation of the immune reaction [12]. Our previous study showed that propofol directly inhibited the viability, proliferation, and invasiveness of HCC cells *in vitro* [13,14]. However, it is unclear whether propofol has a therapeutic effect on HCC *in vivo*.

The role for microRNAs (miRNAs) in carcinogenesis, development and progression of HCC has been well established. Some miRNAs such as miR-122, miR-199 family and MiR-124 is downregulated in HCC and act as putative tumor suppressor genes [15]. Furthermore, overexpression of these miRNAs could induce apoptosis and suppress proliferation of HCC cells [16]. On the contrary, miR-21, miR-221/mir-222 and MiR-224 may act as oncogenes in support of the tumor-promoting activity [17]. In addition, several miRNAs are involved in regulation of the invasive and/or metastatic potential of HCC [18]. Wu et al. has demonstrated that miR-142-3p plays an important role in inhibiting migration and invasion of HCC cells [19]. More importantly, miR-142-3p exerts anti-tumor effect by regulation of the differentiation of macrophages [20]. Thus, modulation of miR-142-3p expression and activity may be a novel approach to HCC therapy.

Microvesicles (MVs), also known as exosomes, are derived from multivesicular endosomes that mediate cell-to-cell communication [21]. Increasing evidence indicated that MVs can shuttle miRNAs into neighboring or distant cells and regulate target gene expression [22]. Yang et al. demonstrated that MVs released from macrophage can transfer miR-123 to breast cancer cells which resulted in promoting metastasis of cancer cells [23]. Therefore, secreted miRNAs may serve as novel therapeutic targets for treatment of cancer. In the present study, we have investigated the effect of propofol on anti-HCC *in vivo*, and demonstrated the underlying mechanisms associated with MVs containing miR-142-3p.

## Method and material

### Chemicals and reagents

RPMI 1640 was obtained from GIBCO (Invitrogen, Carlsbad, CA, USA). Fetal bovine serum (FBS) was obtained from Hyclone (Logan, UT, USA). Lipofectamine 2000 transfection reagent was obtained from Invitrogen Life Technologies (Grand Island, NY, USA). Propofol was purchased from Sigma Aldrich (St Louis, MO, USA). A cholesterol-conjugated miR-142-3p inhibitor and a negative control (Nc) oligonucleotide for in vivo RNA delivery were purchased from RiboBio (Guangzhou, China). Antibodies against RAC1 and GAPDH were purchased from Santa Cruz Biotechnology (Santa Cruz, CA, USA). A detergent-compatible protein assay kit was purchased from Bio-Rad Laboratories (Hercules, CA, USA). An miRNeasy Mini kit, miScript Reverse Transcription kit, and miScript SYBR Green PCR kit were purchased from Qiagen (Hilden, Germany). Clodronate encapsulated in liposome nanoparticles (5 mg/ml) was purchased from Encapsula Nanosciences Inc.

### Cell culture

A mouse HCC cell line, Hepa1-6, and a mouse macrophage cell line, Raw 264.7, were obtained from the Chinese Academy of Sciences (Shanghai, China). The cells were cultured in complete RPMI 1640 with 10% FBS and treated with propofol (dissolved in RPMI 1640) in complete medium. To obtain reliable results, the final concentration of RPMI 1640 in the culture medium was less than 0.1%.

### Animal tumor model

All the experiments were approved by the Experimental Animal Ethics Committee of Zhejiang University and performed under the guidelines of the Institutional Animal Care and Use Committee. Male C57L/J 8-week-old mice were purchased from Shanghai Laboratory Animal Centre (Chinese Academy of Sciences). Hepa1-6 cells (5 × 10^6^ cells) were injected into the right flank of the C57L/J mice for 5 days. The mice were then randomly divided and assigned to the following treatments (*n* = 8 in each group): 20 mg/kg of propofol, 100 μg/kg of clodrolip, 20 mg/kg of propofol + 100 μg/kg of clodrolip, 50 mg/kg of propofol, 50 mg/kg of propofol + 100 μg/kg of clodrolip, 20 μg of MVs, the miR-142-3p inhibitor, or 20 mg/kg of propofol + the miR-142-3p inhibitor. Propofol in 0.5 ml of soybean oil was administered by intraperitoneal (i.p.) injection once a day for 3 weeks. For macrophage depletion, 100 μl of clodronate liposomes were injected via the i.p. route into the tumor-bearing mice weekly. The Nc and the miR-142-3p inhibitor (10 nmol) in 0.1 ml of saline buffer were administered intratumorally every 2 days for 3 weeks. Tumor growth was assessed twice a week with a caliper measurement, and the volume (V) was calculated using the formula V = 1/2 × length × (width)^2^.

### Preparation and culture of mouse tumor-associated macrophages

Tumor-associated macrophages (TAMs) were isolated from the tumors according to previous report [24]. Briefly, small pieces of solid tumors tissue was digested with0.125% (wt/vol) Trypsin (Sigma) for 40 minutes at 37°C, followed by washing twice in incomplete RPMI 1640 medium. Cells were seeded in dishes of RPMI 1640 medium with 10% FBS and, after 12 h of incubation, non-adherent cells were vigorously washed off. The adherent cells were greater than 95% macrophages.

### Isolation of MVs by differential centrifugation

The MVs were isolated from the cell culture medium by differential centrifugation according to the method described in a previous report [21]. The cells and cell debris were removed by centrifugation at 300 × g for 10 min, 1200 × g for 10 min, and 10000 × g for 30 min. Supernatant was sequentially centrifuged at 11,0000 × g for 2 h (all steps were performed at 4°C). The MVs were collected from the pellet and resuspended in phosphate buffer saline (PBS) buffer. The total protein content in the MVs was measured, and the levels of MVs were determined by measuring the total protein content, which is presented as micrograms of total protein in the MVs.

### Generation of recombinant adenoviruses overexpressing RAC1

The full-length coding sequence for mouse RAC1 was amplified by PCR from the mouse full-length cDNA based on GeneBank sequences. DNA encoding the RAC1 was subcloned from pCMV3.0b-myc-RAC1 into pAdTrack-CMV. All the sequences were confirmed by automated DNA sequencing. These shuttle plasmids were then recombined with the backbone vector pAdEasy-1 in BJ5183 bacteria. Viral particles were purified using the Virabind™ Adenovirus Purification Kit (Cell Biolabs, Inc., San Diego, CA, USA). Raw 264.7 cells were infected with adenovirus at an MOI of 100 at 37°C for 2 h. The cells were then cultured in fresh medium for 24 h or 48 h.

### Oligonucleotides and Cell Transfection

Hepa1-6 and Raw 264.7 cells were seeded in 6-well or 24-well plates and transfected using Lipofectamine 2000 (Invitrogen, Carlsbad, CA, USA) according to the manufacturer’s instructions. For the knockdown of miR-142-3p, miR-142-3p inhibitor or a negative-control (Nc) was used at the concentration of 100 nM. The cells were treated with propofol after the transfection for 24 h.

### Quantitative real-time PCR (Q-PCR) analysis

Approximately 5 × 10^6^ cells were treated with or without (control) propofol for 24 h. Mature miRNAs were isolated and purified using Trizol reagent (Invitrogen, Carlsbad, CA, USA), according to the manufacturer’s protocol. The levels of miR-142-3p, miR-199a, miR-122, and miR-29 were quantified with a TaqMan PCR kit. Real-time PCR was performed with LightCycler 480, using U6 small nuclear RNA as an internal normalized reference.

The expression of mRNA was measured using SYBR GREEN PCR Master Mix (Applied Biosystems). The specific primers were as follows: RAC1, 5′-TTGCCAAAATACCTTCTGAACT-3′ (forward) and 5′-TGCTTTACGCATCTGAGAACTA-3′ (reverse); GAPDH, 5′-TGAAGCAGGCA TCTGAGGG-3′ (forward) and 5′-CGAAGGTGGAAGAGTGGGAG-3′ (reverse). All data were analyzed using GAPDH gene expression as an internal standard.

### Tumor invasion assay

The tumor invasion assay used modified Boyden chambers (8-μm pore size, BD Biosciences, USA), in which the various cellular components were grown without direct cell-to-cell contact. Briefly, the membrane was coated with Matrigel (BD) diluted 1/5 in serum-free DMEM media on ice. Hepa1-6 cells (1 × 10^5^ cells) were seeded in the upper well of the chamber, and the lower well was filled with TAMs. After co-culture for 24 h, the transwells were removed from the 24-well plates, and the non-invaded cells were scraped off the top of the Transwell filters with a cotton swab. The filter was fixed formaldehyde (4%) and stained with 0.1% crystal violet in 2% methanol. Invasiveness was determined by counting cells in five microscopic fields per well, and the extent of invasion was expressed as an average number of cells per microscopic field.

### Statistical analysis

Statistical analysis was performed with SPSS 13.0 software. The statistical analyses used an analysis of variance (ANOVA) or the Student’s *t*-test. Data were expressed as the mean ± standard deviation. *P* < 0.05 was considered significant.

## Results

### Propofol inhibited HCC growth through macrophage *in vivo*

**Figure 1:**
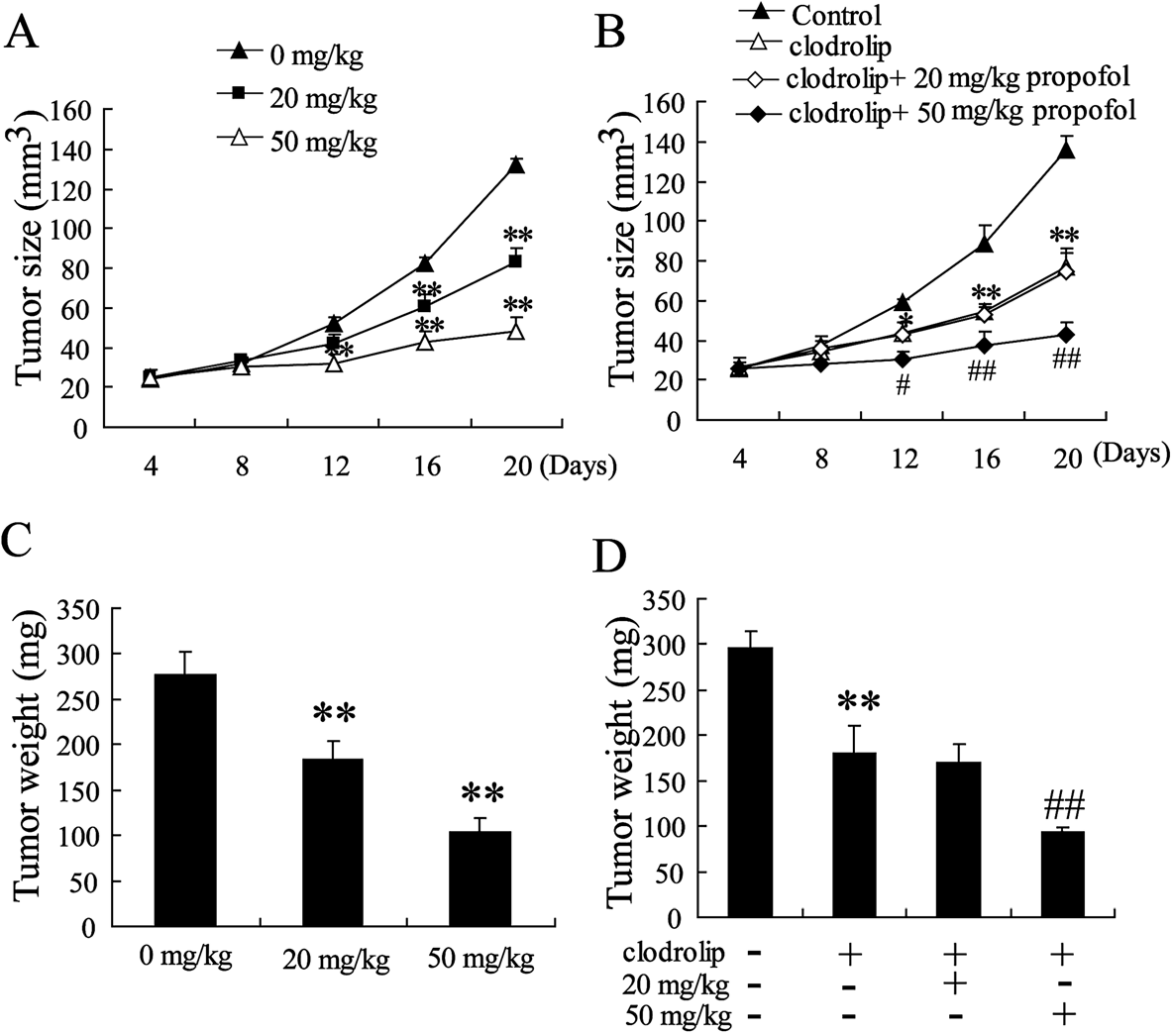
**Propofol inhibited HCC growth** ***in vivo*** **.** The tumor-bearing mice were treated with 0 mg/kg, 20 mg/kg and 50 mg/kg propofol by i.p. injection. **(A)**Tumor sizes on each mouse were monitored 2 times per week and **(C)** tumor weights were measured. The tumor-bearing mice were treated with 100 μg/kg clodrolip, 20 mg/kg propofol + 100 μg/kg clodrolip, 50 mg/kg propofol + 100 μg/kg clodrolip, **(B)** Tumor sizes and **(D)** tumor weights were determined. *P < 0.05, **P < 0.01 indicate significant differences from control group. ^#^P < 0.05, ^##^P < 0.01 indicate significant differences from clodrolip group.

Our previous study showed that propofol induced apoptosis and impaired the invasiveness of HCC cell lines *in vitro* [14,15]. To evaluate the effects of propofol on HCC in vivo, we examined tumor growth in tumor-bearing mice treated with propofol. The size of the tumors significantly decreased in a dose-dependent manner following the i.p. injection of 20 and 50 mg/kg of propofol (Figure 1A). The tumor-bearing mice were sacrificed on Day 26 after the injection of the Hepa1-6 cells. The mean tumor volume, which was 276.67 ± 25.17 mm^3^ in the control group, was dose-dependently reduced in the propofol treatment group (183.33 ± 20.83 mm^3^ in the 20 mg/kg group and 103.33 ± 15.28 mm^3^ in the 50 mg/kg group) (Figure 1B).

To determine whether the administration of propofol elicited the activation of macrophages within a HCC environment, we analyzed the expression of TNF-αand CD206 in the TAMs of the hepatoma-bearing mice injected with different amounts of propofol. The TAMs from the propofol-injected mice had higher expression of TNF-α but lower expression of CD206 (Figure 1C and D).

### Cocultivation with TAMs from the propofol–treated HCC tissue increased miR-142-3p levels in HCC cells

**Figure 2:**
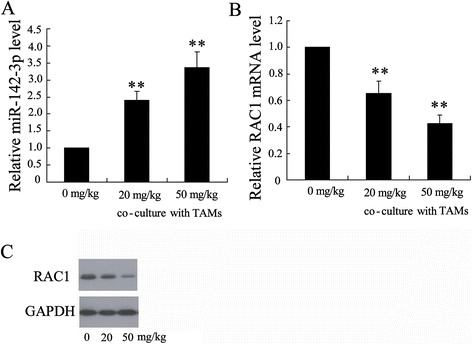
**Co-cultivation with TAMs from propofol–treated HCC tissue increased miR-142-3p levels in HCC cells. (A)** The expression of miR-142-3p in Hepa1-6 cells after 24 h co-culture with TAMs isolated from tumor-bearing mice treated with 0 mg/kg, 20 mg/kg and 50 mg/kg propofol by i.p. injection. The relative mRNA **(B)** and protein **(C)** levels of RAC1 in Hepa1-6 cells after 24 h co-culture with TAMs isolated from tumor-bearing mice treated with 0 mg/kg, 20 mg/kg and 50 mg/kg propofol by i.p. injection. **P < 0.01, indicate significant differences from TAMs isolated from tumor-bearing mice treated with 0 mg/kg propofol.

To explore the mechanism underling the involvement of TAMs in the antitumor activity of propofol, we measured the expression of a number of miRNAs, including miR-142-3p, miR-199a, miR-122, and miR-29, which may act as tumor suppressors in HCC [23]. Hepa1-6 cells cocultured with TAMs (1:1) from the propofol-treated HCC tissue exhibited a dose-dependent increase in cellular miR-142-3p levels relative to cells that were cocultured with TAMs from soybean oil-treated HCC tissue (Figure 2A). However, there was no change in the expression of miR-199a, miR-122, and miR-29 (Figure 2B). The expression of RAC1, a known target of miR-142-3p [25], was significantly down-regulated in the HCC cells following incubation with TAMs from the propofol-treated HCC tissue (Figure 2C and 2D), suggesting that the level of functional miR-142-3p levels increased in the HCC cells.

### The administration of propofol stimulated miR-142-3p shuttling from macrophages to HCC cells

**Figure 3:**
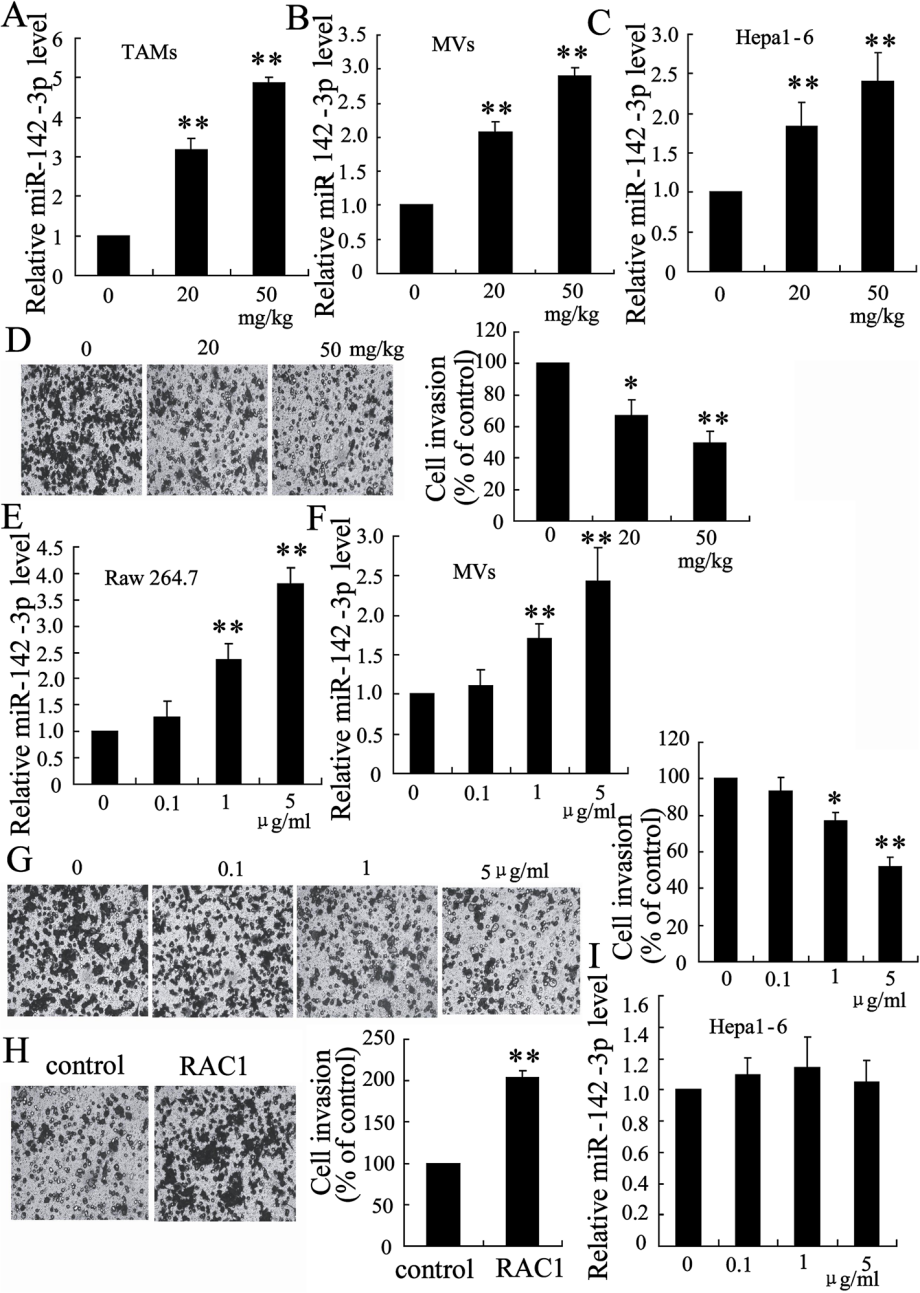
**Propofol stimulated miR-142-3p shuttling from macrophages to HCC cells.** The level of miR-142-3p was determined **(A)** in TAMs from tumor-bearing mice injected with propofol, **(B)** in MVs from propofol-treated TAMs and **(C)** in Hepa1-6 cells treated with MVs from propofol-treated TAMs. **(D)** Hepa1-6 cells were co-cultured with TAMs for 48 h. Cell invasiveness was measured using a transwell invasion assay. The invasion of Hepa1-6 cells was assessed by counting the cells in the basolateral side of the transwell filters under a light microscope. The level of miR-142-3p was determined **(E)** in Raw 264.7 cells treated with different concentration of propofol for 24 h and **(F)** in MVs from Raw 264.7 cells. **(G)** Hepa1-6 cells were incubated with MVs from propofol-treated Raw 264.7 cells for 48 h. The invasion of Hepa1-6 cells was assessed. **(H)** After transfection of RAC1 expressing plasmid for 24 h, Hepa1-6 cells were treated with MVs from propofol-treated Raw 264.7 cells for 48 h. The invasion of Hepa1-6 cells was assessed. **(I)** The level of miR-142-3p was measured in Hepa1-6 cells treated with different concentration of propofol for 24 h. *P < 0.05 and **P < 0.01, indicate significant differences from control.

A previous study demonstrated that miR-142-3p prevents the differentiation of macrophages and inhibits their immunosuppressive function in tumors [24]. To determine whether propofol treatment stimulated miR-142-3p shuttling from macrophages to the HCC cells, we determined the expression of miR-142-3p in macrophages and MVs secreted from macrophages. As expected, the expression of miR-142-3p in the TAMs of the tumor-bearing mice injected with propofol was obviously increased compared with those of the soybean oil-injected mice (Figure 3A). Consistent with this observation, the level of miR-142-3p in the MVs from the propofol-treated TAMs was also higher than that in the soybean oil-treated mice (Figure 3B). The levels of miR-142-3p were significantly increased in the Hepa1-6 cells incubated with the MVs of the propofol-treated TAMs (Figure 3C). Furthermore, the migration of Hepa1-6 cells was significantly decreased (Figure 3D).

We further analyzed the effect of propofol on the expression of miR-142-3p in macrophages and MVs *in vitro*. Briefly, the Raw 264.7 cells were exposed to different concentrations of propofol for 24 h. The levels of miR-142-3p were dose-dependently elevated in both macrophages and MVs (Figure 3E and 3F). The incubation with the MVs from the propofol-treated Raw 264.7 cells significantly inhibited the migration of Hepa1-6 cells (Figure 3G). Overexpression of RAC1 in Hepa1-6 cells reversed the effect of these MVs on the migration of the HCC cells (Figure 3H). However, the expression of miR-142-3p was not up-regulated when the Hepa1-6 cells were directly treated with propofol (Figure 3I).

### MVs in the plasma of the propofol-injected mice enhanced anti-HCC activity *in vivo*

**Figure 4:**
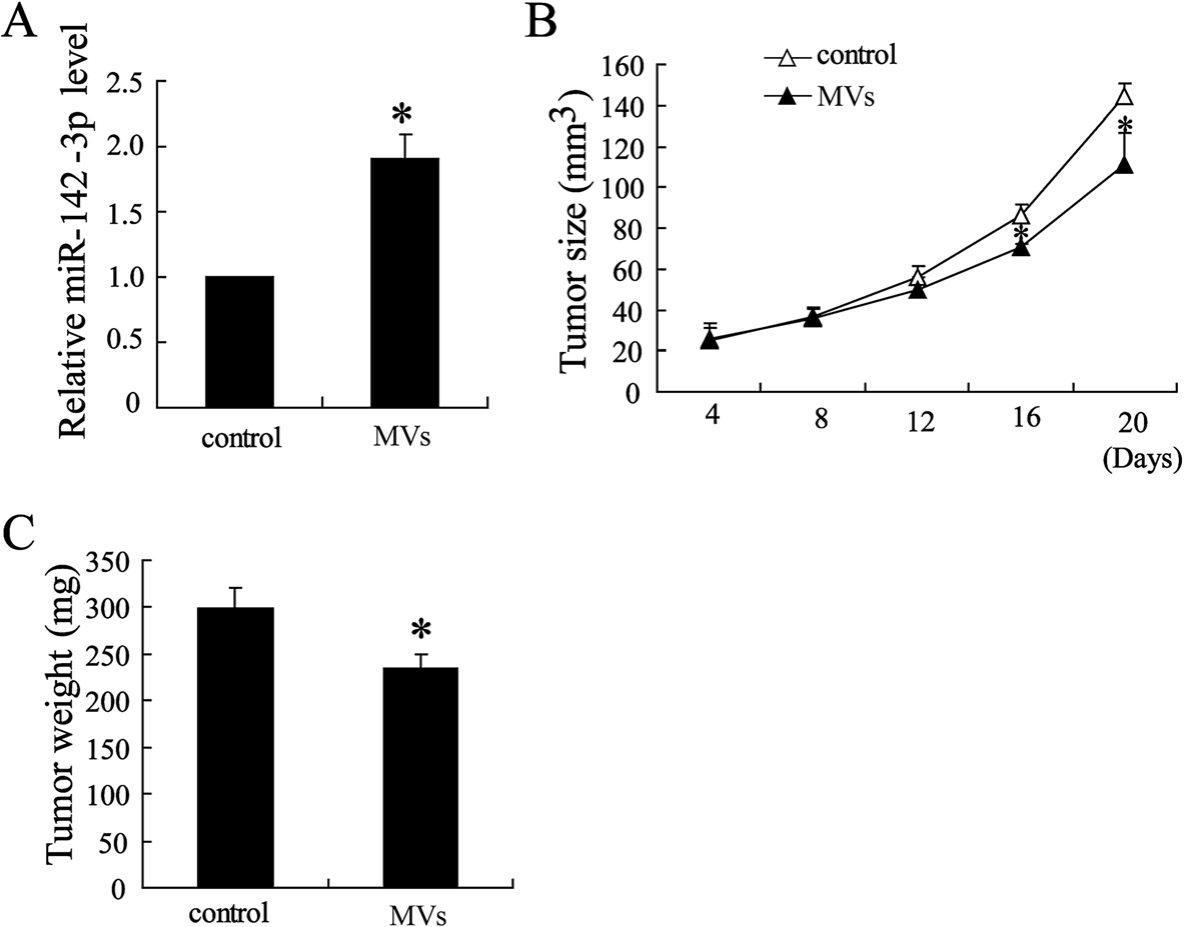
**MVs collected from the plasma of tumor-bearing mice injected with propofol enhance anti-HCC** ***in vivo*** **.** The tumor-bearing mice were treated with MVs collected from the plasma of tumor-bearing mice injected with or without propofol. **(A)** The level of plasma miR-142-3p was detected. **(B)** Tumor sizes and **(C)** tumor weights were measured in tumor-bearing mice injected with plasma MVs. *P < 0.05, indicate significant differences from control.

To further explore the role of MVs in anti-HCC, we collected MVs from the plasma of the tumor-bearing mice injected with or without propofol (20 mg/kg). Plasma miR-142-3p levels were increased in the tumor-bearing mice treated with the MVs of the propofol-injected mice (Figure 4A). Tumor growth was also significantly inhibited in these mice (Figure 4B and 4C). These results suggest that secreted miR-142-3p may be involved in the anti-HCC effect of propofol.

### miR-142-3p was involved in the anti-tumor activity of propofol

**Figure 5:**
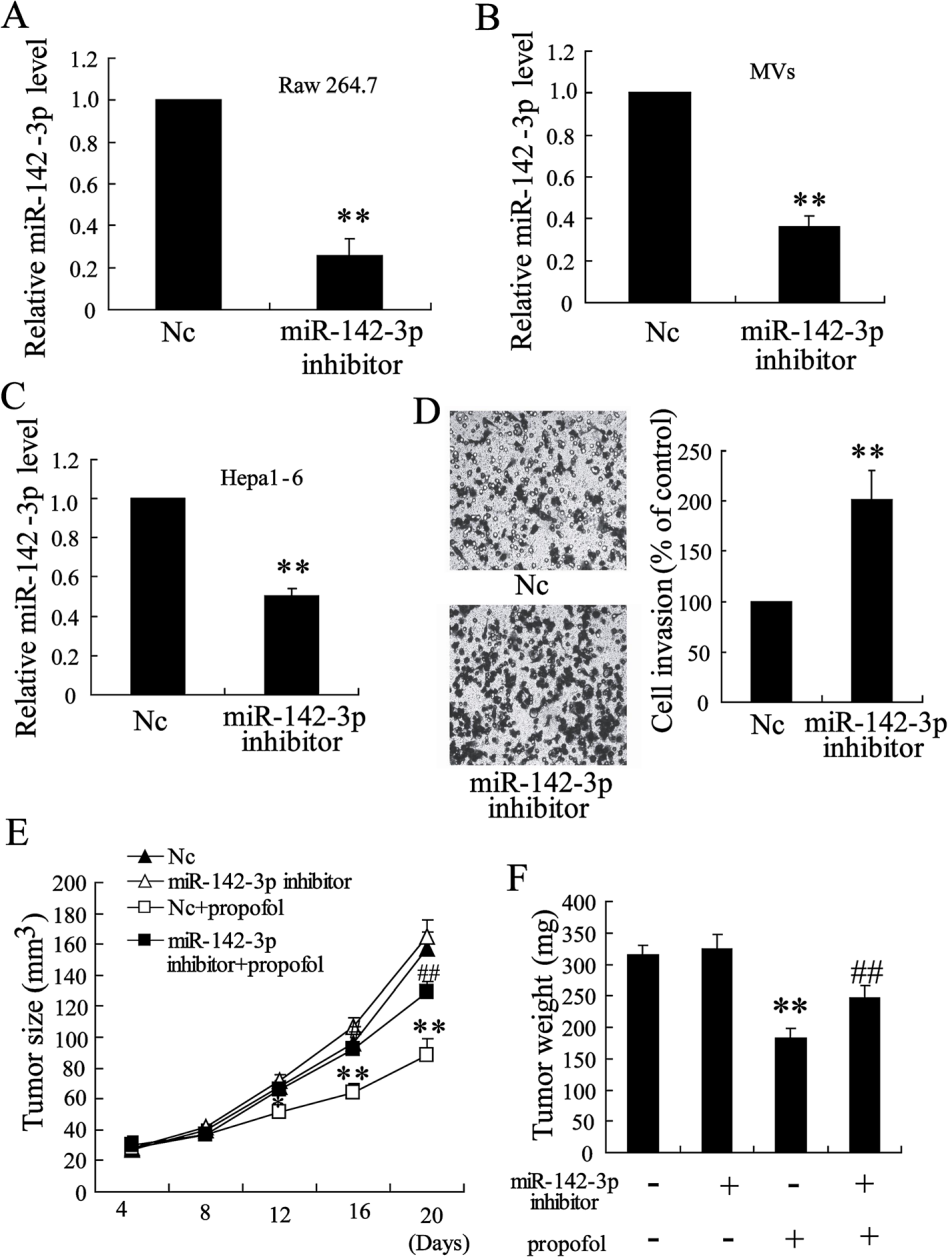
**miR-142-3p was involved in the effect of propofol on anti-tumor activity. (A)** The expression of miR-142-3p was detected in Raw 264.7 cells transfected with miR-142-3p inhibitor. **(B)** The expression of miR-142-3p was measured in MVs from Raw 264.7 cells transfected with miR-142-3p inhibitor. The expression of miR-142-3p **(C)** and the invasion ability **(D)** were detected in Hepa1-6 cells treated with MVs from Raw 264.7 cells transfected with Nc or miR-142-3p inhibitor. **(E)** Tumor sizes and **(F)** tumor weights were measured in tumor-bearing mice injected with Nc or miR-142-3p inhibitor. *P < 0.05, **P < 0.01 indicate significant differences from control group. ^#^P < 0.05, ^##^P < 0.01 indicate significant differences from propofol group.

To further explore the role of miR-142-3p in MVs from macrophages, we treated Raw 264.7 cells with an miR-142-3p inhibitor. The results of real-time PCR showed that the miR-142-3p inhibitor profoundly reduced the levels of miR-142-3p in the Raw 264.7 cells and MVs (Figure 5A and 5B). The MVs from the propofol-treated cells (5 μg/ml) that were transfected with the miR-142-3p inhibitor significantly decreased miR-142-3p levels in Hepa1-6 cells and stimulated the migration of these cells (Figure 5C and 5D).

After an intratumoral injection of the miR-142-3p inhibitor, the size of the tumors in the tumor-bearing mice slightly increased. However, the inhibition of tumor growth by propofol (20 mg/kg) was restored by the miR-142-3p inhibitor (Figure 5E and 5F). These results indicate that miR-142-3p was involved in the anti-HCC activity of propofol.

## Discussion

This is the first study describing the anti-HCC effect of propofol *in vivo*. We demonstrated that propofol inhibited tumor growth in tumor-bearing mice by activation of macrophages. Propofol stimulated TAMs to secrete MVs and deliver miR-142-3p to HCC cells, resulting in the inhibition of HCC cell invasion. In addition, MVs collected from the plasma of the tumor-bearing mice injected with propofol suppressed tumor growth. More importantly, down-regulation of miR-142-3p expression reversed the effect of propofol on HCC cell migration.

Tumor-associated macrophages (TAMs) promote tumor cell proliferation, invasion, and angiogenesis [26]. In this study, we first demonstrated that macrophages may serve as treatment targets of propofol. The deletion of macrophage activation using clodrolip abolished the anti-HCC activity of a moderate dose of propofol (20 mg/kg). The combination of a high dose of propofol (50 mg/kg) and clodrolip was less than twice as effective as either alone in inhibiting tumor growth. Our previous study also showed that propofol directly induced apoptosis and inhibited the invasiveness of HCC cells [13,14]. Therefore, a low or moderate dosage of propofol exerts anti-HCC activity mainly through macrophages, whereas a high dose of propofol inhibits tumor growth *via* macrophages and HCC cells.

Within the tumor microenvironment, MVs mediate communication between macrophages and tumor cells [27,28]. We demonstrated that propofol exerted anti-HCC activity by modulating the expression of miR-142-3p in macrophage-derived MVs. First, miR-142-3p plasma levels were increased in the MVs of the propofol-injected mice, and this significantly inhibited tumor growth. Second, the administration of MVs from propofol-treated macrophages increased miR-142-3p levels in HCC cells, but propofol itself did not up-regulate miR-142-3p expression in HCC cells. Third, MVs shed from propofol-injected TAMs were enriched in miR-142-3p. By depleting miR-142-3p expression, we showed that the miR-142-3p in the MVs accounted for the anti-HCC effect of propofol. We demonstrated that the miR-142-3p in the MVs was taken up by HCC cells and subsequently inhibited cell migration.

A previous study confirmed that miR-142-3p suppressed migration and invasion of HCC cells through down-regulation of RAC1 [19]. As a target of miR-142-3p, RAC1 plays a key role in tumor cell growth, migration and invasion [29,30]. We demonstrated that the expression of RAC1 was significantly decreased in HCC cells following incubation with TAMs from propofol–treated HCC tissue. More importantly, overexpression of RAC1 in HCC cells could abolish the effect of MVs on the migration of tumor cells.

## Conclusions

In summary, we demonstrated that propofol effectively inhibited tumor growth *in vivo*. We showed that propofol stimulated miR-142-3p shuttling from macrophages to HCC cells and that miR-142-3p down-regulated RAC1 expression, thus, inhibiting HCC cell migration and invasion.

## References

[1] Gomes MA, Priolli DG, Tralhão JG, Botelho MF (2013). Hepatocellular carcinoma: epidemiology, biology, diagnosis, and therapies. Rev Assoc Med Bras.

[2] Herszényi L, Tulassay Z (2010). Epidemiology of gastrointestinal and liver tumors. Eur Rev Med Pharmacol Sci.

[3] Wang J, He XD, Yao N, Liang WJ, Zhang YC (2013). A meta-analysis of adjuvant therapy after potentially curative treatment for hepatocellular carcinoma. Can J Gastroenterol.

[4] Chan DL, Morris DL, Chua TC (2013). Clinical efficacy and predictors of outcomes of repeat hepatectomy for recurrent hepatocellular carcinoma - a systematic review. Surg Oncol.

[5] Gluer AM, Cocco N, Laurence JM, Johnston ES, Hollands MJ, Pleass HC, Richardson AJ, Lam VW (2012). Systematic review of actual 10-year survival following resection for hepatocellular carcinoma. HPB (Oxford).

[6] Zhou XD (2002). Recurrence and metastasis of hepatocellular carcinoma: progress and prospects. Hepatobiliary Pancreat Dis Int.

[7] Ren XF, Li WZ, Meng FY, Lin CF (2010). Differential effects of propofol and isoflurane on the activation of T-helper cells in lung cancer patients. Anaesthesia.

[8] Miyata T, Kodama T, Honma R, Nezu Y, Harada Y, Yogo T, Hara Y, Tagawa M (2013). Influence of general anesthesia with isoflurane following propofol-induction on natural killer cell cytotoxic activities of peripheral blood lymphocytes in dogs. J Vet Med Sci.

[9] Mammoto T, Mukai M, Mammoto A, Yamanaka Y, Hayashi Y, Mashimo T, Kishi Y, Nakamura H (2002). Intravenous anesthetic, propofol inhibits invasion of cancer cells. Cancer Lett.

[10] Tsuchiya M, Asada A, Arita K, Utsumi T, Yoshida T, Sato EF, Utsumi K, Inoue M (2002). Induction and mechanism of apoptotic cell death by propofol in HL-60 cells. Acta Anaesthesiol Scand.

[11] Miao Y, Zhang Y, Wan H, Chen L, Wang F (2010). GABA-receptor agonist, propofol inhibits invasion of colon carcinoma cells. Biomed Pharmacother.

[12] Inada T, Kubo K, Shingu K (2011). Possible link between cyclooxygenase-inhibiting and antitumor properties of propofol. J Anesth.

[13] Zhang J, Zhang D, Wu GQ, Feng ZY, Zhu SM (2013). Propofol inhibits the adhesion of hepatocellular carcinoma cells by upregulating microRNA-199a and downregulating MMP-9 expression. Hepatobiliary Pancreat Dis Int.

[14] Zhang J, Wu GQ, Zhang Y, Feng ZY, Zhu SM (2013). Propofol induces apoptosis of hepatocellular carcinoma cells by upregulation of microRNA-199a expression. Cell Biol Int.

[15] Hung CH, Chiu YC, Chen CH, Hu TH (2014). MicroRNAs in hepatocellular carcinoma: carcinogenesis, progression, and therapeutic target. Biomed Res Int.

[16] Sun J, Lu H, Wang X, Jin H (2013). MicroRNAs in hepatocellular carcinoma: regulation, function, and clinical implications. Sci World J.

[17] Li W, Xie L, He X, Li J, Tu K, Wei L, Wu J, Guo Y, Ma X, Zhang P, Pan Z, Hu X, Zhao Y, Xie H, Jiang G, Chen T, Wang J, Zheng S, Cheng J, Wan D, Yang S, Li Y, Gu J (2008). Diagnostic and prognostic implications of microRNAs in human hepatocellular carcinoma. Int J Cancer.

[18] Gailhouste L, Ochiya T (2013). Cancer-related microRNAs and their role as tumor suppressors and oncogenes inhepatocellular carcinoma. Histol Histopathol.

[19] Wu L, Cai C, Wang X, Liu M, Li X, Tang H (2011). MicroRNA-142-3p, a new regulator of RAC1, suppresses the migration and invasion of hepatocellular carcinoma cells. FEBS Lett.

[20] Sonda N, Simonato F, Peranzoni E, Calì B, Bortoluzzi S, Bisognin A, Wang E, Marincola FM, Naldini L, Gentner B, Trautwein C, Sackett SD, Zanovello P, Molon B, Bronte V (2013). miR-142-3p prevents macrophage differentiation during cancer-induced myelopoiesis. Immunity.

[21] Muralidharan-Chari V, Clancy JW, Sedgwick A, D’Souza-Schorey C (2010). Microvesicles: mediators of extracellular communication during cancer progression. J Cell Sci.

[22] Morello M, Minciacchi VR, de Candia P, Yang J, Posadas E, Kim H, Griffiths D, Bhowmick N, Chung LW, Gandellini P, Freeman MR, Demichelis F, Di Vizio D (2013). Large oncosomes mediate intercellular transfer of functional microRNA. Cell Cycle.

[23] Yang M, Chen J, Su F, Yu B, Su F, Lin L, Liu Y, Huang JD, Song E (2011). Microvesicles secreted by macrophages shuttle invasion-potentiating microRNAs intobreast cancer cells. Mol Cancer.

[24] Zhang Y, Liu D, Chen X, Li J, Li L, Bian Z, Sun F, Lu J, Yin Y, Cai X, Sun Q, Wang K, Ba Y, Wang Q, Wang D, Yang J, Liu P, Xu T, Yan Q, Zhang J, Zen K, Zhang CY (2010). Secreted monocytic miR-150 enhances targeted endothelial cell migration. Mol Cell.

[25] Hara M, Kono H, Furuya S, Hirayama K, Tsuchiya M, Fujii H: **Macrophage colony-stimulating factor plays a pivotal role in chemically induced hepatocellular carcinoma in mice.** *Hepatol Res* 2013, [Epub ahead of print]. 10.1111/hepr.12174 23710613

[26] Yuan A, Chen JJ, Yang PC (2008). Pathophysiology of tumor-associated macrophages. Adv Clin Chem.

[27] D’Souza-Schorey C, Clancy JW (2012). Tumor-derived microvesicles: shedding light on novel microenvironment modulators and prospective cancer biomarkers. Genes Dev.

[28] Antonyak MA, Cerione RA (2014). Microvesicles as mediators of intercellular communication in cancer. Methods Mol Biol.

[29] Wu J, Meng J, Du Y, Huang Y, Jin Y, Zhang J, Wang B, Zhang Y, Sun M, Tang J (2013). RACK1 promotes the proliferation, migration and invasion capacity of mouse hepatocellular carcinoma cell line *in vitro* probably by PI3K/Rac1 signaling pathway. Biomed Pharmacother.

[30] Yang W, Lv S, Liu X, Liu H, Yang W, Hu F (2010). Up-regulation of Tiam1 and Rac1 correlates with poor prognosis in hepatocellular carcinoma. Jpn J Clin Oncol.

